# Supplementary material for: Footprint of sustained poleward warm water flow within East Antarctic submarine canyons
Source: Nat Commun. 2024 Jul 17;15:6028. doi: 10.1038/s41467-024-50160-z (PMC11254908; doi:10.1038/s41467-024-50160-z)
Supplement: Supplementary file 1 — Supplementary Information [file 41467_2024_50160_MOESM1_ESM.pdf]

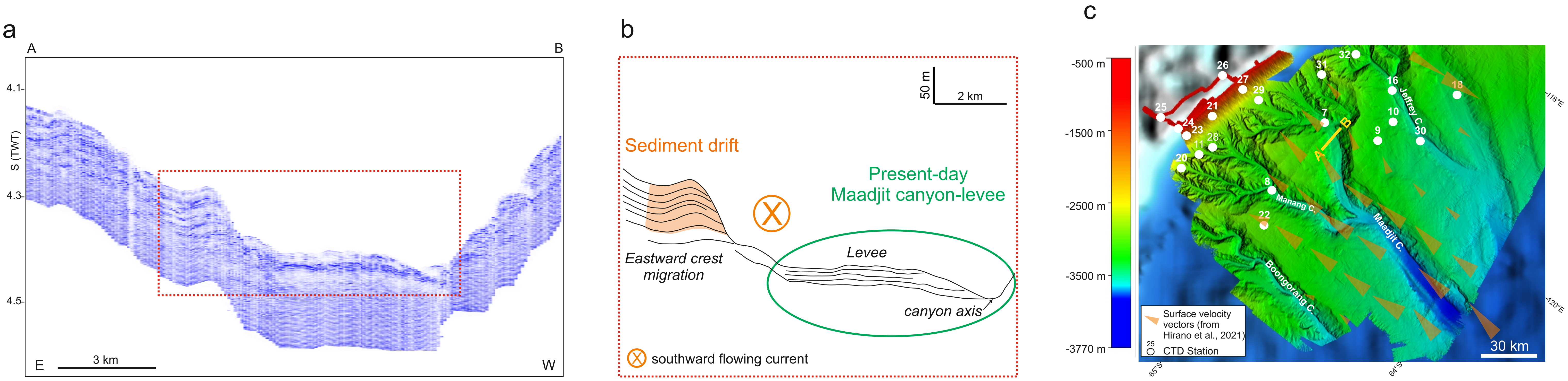

Supplementary Figure 1. a. Part of the sub-bottom CHIRP profile 76 collected on the Sabrina Coast continental rise; b. Seismostratigraphic interpretation highlighting the relationship between the sediment drift on the eastern flank of the Maadjit Canyon and the coeval or even older levee deposit to the west of it; c. Location map of the sub-bottom CHIRP together with the path of currents carrying CDW toward the continental shelf (light orange arrows; Hirano et al., 2021).

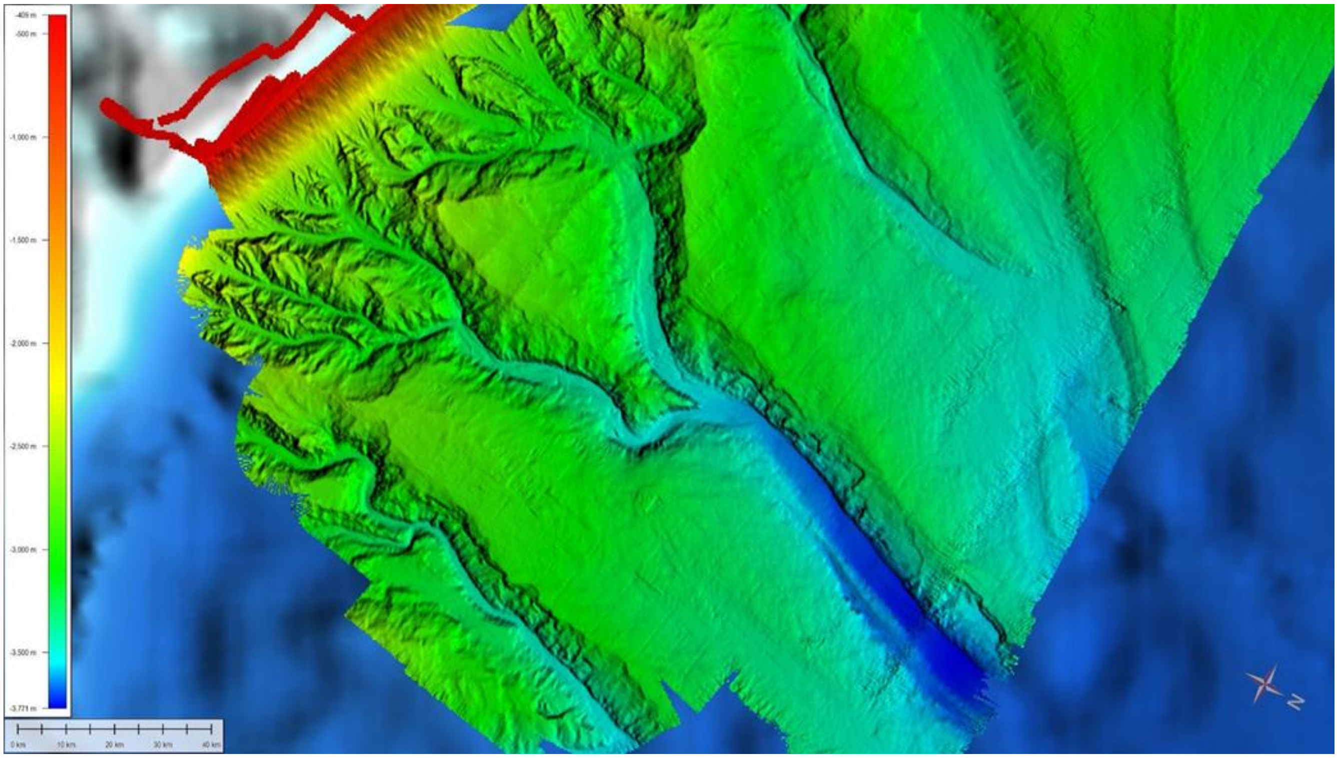

Supplementary Figure 2. Part of the un-interpreted multibeam bathymetry collected off Sabrina Coast
